# Use of inhaled versus oral steroids for acute dysphonia

**DOI:** 10.5935/1808-8694.20130035

**Published:** 2015-11-02

**Authors:** Andréa Moreira Veiga de Souza, André de Campos Duprat, Rejane Cardoso Costa, Janaína de Oliveira Pimenta, Fernanda Fonseca de Sá Andrade, Fernanda Ferreira da Silva

**Affiliations:** a MD MSc - Otorhinolaryngologist - Espaço da voz - MG - Brazil; b MD PhD - Otorhinolaryngologist. Professor at the Medical School of the Santa Casa - São Paulo; c MD Otorhinolaryngologist - Insttuto de Otorrinolaringologia de Minas Gerais - Brazil; d MSc - Speech and Hearing Therapist - Espaço da voz - MG - Brazil. Irmandade da Santa Casa de Misericórdia de São Paulo

**Keywords:** adrenal cortex hormones, dysphonia, laryngitis, treatment outcome

## Abstract

Acute dysphonia is a frequent condition in clinical practice. Its treatment, especially in adults, is not well established in the literature. Steroids are the most recommended drug treatment. However, the existing studies are not enough to establish superiority among the different steroids and the best route of administration.

**Objective:**

This prospective clinical study aimed at comparing the effect of inhaling steroids as a dry powder with the effect of oral steroids to treat acute dysphonia.

**Method:**

We assessed 32 adult patients, broken down into two groups of 16 patients in each one of the treatments, before and seven days after the use of the medication. The patients were submitted to videolaryngoscopy and perceptive and acoustic voice assessment.

**Result:**

Oral and inhalation treatment significantly reduced hyperemia and edema, and improved the muco-ondulatory movement; nonetheless, edema reduction was statistically more significant (*p* = 0.012) in the patients treated with the inhalation form of the drug. However, comparing the values of the auditory perceptive analysis and the acoustic measures after treatment between the groups was not statistically significant.

**Conclusion:**

There was a significant improvement in the acute laryngitis concerning the assessments carried out in all the patients assessed, concerning the two treatments. The inhalation steroid treatment was significantly more effective in reducing the edema.

## INTRODUCTION

Acute dysphonia impacts verbal communication and it may lead to numerous types and levels of limitations which have repercussions both in the individual's social as well as professional lives. In such a case, it is increasingly more frequent in clinical practice to have patients who seek medical care, eager for a rapid and effective intervention, which enables voice normalization and return to their activities as soon as possible.

Acute dysphonia may stem from various factors, such as: inflammatory processes, trauma, laryngeal paralysis, psychological factors, and others. Laryngitis, inflammation of laryngeal tissues, basically characterized by edema and/or hyperemia, is the most frequent finding in the clinical exam of these patients^1^, ^2^, ^3^. In the acute and subacute forms of the disease, onset is usually sudden and the disease lasts for less than three weeks^4^.

Vocal rest is paramount in the treatment of acute dysphonia by laryngitis^2^; however, for most of the patients who came for medical care it is not possible to do it, other treatments are needed.

Steroid treatment is described as fundamental in the treatment of acute laryngitis, especially when there is breathing compromise in children and voice involvement in adults^5^, ^6^, ^7^, ^8^, ^9^. The goal is to reduce inflammation, relieve the pain and reestablish mucosal physiology. Notwithstanding, existing studies are not enough to establish which steroids are better and which are the best means of administration.

Oral steroids have a powerful anti-inflammatory effect and their indication in inflammatory diseases and laryngeal edema is well know^5^, ^7^, ^8^, ^10^. When used for short periods of time (up to two weeks), they have a very low likelihood of causing side effects. Oral steroids are as effective as the injectable ones, which speaks against the use of injectable steroids when the oral administration is possible^11^.

Today, topical steroids are largely used as the treatment of choice for inflammatory processes of the airways, such as rhinitis and asthma. They have a powerful anti-inflammatory action and in reducing edemas. Since they act directly on the inflammation site, they can be used in lower doses. In shorter treatment courses, they have fewer side effects and a better safety profile than systemic steroids^12^, ^13^. They are indicated to be used in laryngeal disorders, such as in the treatment of children with croup and laryngotracheitis, and in laryngeal edema - in cases of post-endotracheal laryngeal intubation edema. However, here are no reports of its use in acute laryngitis in adults.

Some studies have described cases of dysphonia and laryngeal lesions in adult patients under chronic use of steroids and other inhalation medication to treat asthma, as well as laryngeal irritation, cough and oropharyngeal candidiasis^14^, ^15^, ^16^, ^17^, ^18^, ^19^, ^20^. The lesions described are associated with numerous factors, such as medication dose, duration of use, type of inhaler and propellant and other drugs present in the medication, cough, gastroesophageal reflux, and/or associated smoking and disease, or degree of inflammatory process in the airway^17^. In all these studies, the patients were under use of the inhalation medication for at least two weeks, and the dysphonia and lesions were reversible after interrupting the medication.

Based on the side effects, some authors do not recommend the use of inhalation steroids in people who make professional use of their voices^2^, ^19^. This may justify the fact that in the literature there is only one paper showing the effectiveness of inhalation steroids in acute laryngitis^20^.

This lack of studies published, coupled to the demand for cases of adult patients with dysphonia associated with acute laryngitis, to the repercussion of the acute loss of voice in one's social and professional lives and the need for standardization and normalizing of the existing treatments, motivated the development of this study.

This study aimed at assessing and comparing the effects of inhalation steroids (inhalation fluticasone) as a dry powder, with the effects of oral steroids (prednisone) in the treatment of dysphonia associated with acute laryngitis.

## METHOD

This methodology was based on the protocol proposed by Dejonckere et al.^20^ for the functional assessment of voices with disorders and their treatment, and it was approved by the Ethics Committee, project #501/07.

Between January of 2007 and September of 2008 we selected 32 adult patients with acute dysphonia caused by laryngitis, seen in a private clinic - specialized in professional use of the voice. We took off the study: smokers, people with mental disorders, those with psychological and motor problems; those with laryngeal paralysis or structural disorders, or those with a past of laryngeal surgery; patients under use of anti-inflammatory medication or drugs to treat gastric reflux. Those individuals with hypersensitivity to fluticasone or prednisolone, or contraindication concerning their use were taken off, as well as those with indications for using other drugs, such as antibiotics, anti-coughing medication, antipyretic or expectorants, to treat laryngitis. We took off the study those patients with vocal fold hematoma, because vocal rest in these cases would be mandatory and vocal rest was not a specific recommendation given to these patients.

The patients were randomly broken down into two groups; the first group received 50 mcg of inhalation fluticasone, twice-a-day, and the second group received 20 mg of oral prednisolone, twice-a-day, both for 7 days. No patient was instructed to perform vocal rest. All the patients maintained their vocal activities.

All the patients were assessed by videolaryngostroboscopy, perceptive analysis (GRBAS) and voice acoustics on the first and seventh (last) day of treatment. At the end of treatment (7^th^ day), all of them were asked to answer a questionnaire.

The inhaled steroid chosen was fluticasone, since it was described as the most powerful inhaled steroid, the one remaining the longer on the mucosa and the one with the least systemic absorbing^13^, ^21^, ^22^. Administration of the inhaled dry powder, for it is the one with the least likelihood of causing larynx damage^22^, ^23^ and the dose of 100 mcg/day, for being the lowest effective dose and without reports of local or systemic side effects with this dose in a short-duration treatment.

Prednisolone was chosen because it is one of the most utilized systemic steroids, it has an intermediate duration half-life and causes less side effects^12^. The 40 mg/day dose was the mean value of the doses recommended in other studies^2^, ^3^, ^6^, ^7^, ^8^.

### Videolaryngostroboscopy assessment

Videolaryngostroboscopy, with a JC Biocam laryngoscope was always carried out by the same otorhinola-ryngologist, on the first and 7^th^ day.

These tests were saved using a Philips DVD recorder, for recording and later analysis of the following information: presence or absence of vocal fold hyperemia, presence or absence of vocal fold edema and vocal fold muco-undulatory movement (MOM): normal or changed. Any alteration in the regularity and/or symmetry of the MOM was considered.

### Vocal assessment

After doing the videolaryngostroboscopy exam, a speech and hearing therapist recorded the voice.

The voice was recorded in a silence room, with noise levels below 50 db, by means of a Shure SM 10A, condensed unidirectional microphone, positioned at 45^o^ and at 5 cm away from the user's mouth. It was then connected to an Eurorack UB502 sound board, directly attached to a Pentium 5 Toshiba laptop with a Sound Blaster 32 soundboard from creative labs. The patient was asked to perform the following phonation activities:

• Utter the vowel [a] three times, as long as possible (sustained in one single exhalation), in a habitual tone to measure the maximum phonation time and for a perceptive assessment utilizing the GRBAS scale.
• Utter the sustained vowel [e] for acoustic analysis purposes.

For such acoustic analysis, aiming at doing a quantitative analysis of the sound, we utilized the DR Speech software, version 3.1 and measured the mean values of the fundamental frequency (Fo), jitter, shimmer, neutralized noise energy (NNE) and the harmonic noise ratio (HNR), utilizing a middle stretch of the sustained vowel [e] uttered - such vowel is the basis of the DR Speech program.

### Questionnaire

On the 7^th^ day of treatment, the patients from both groups were asked to answer a written questionnaire, reporting whether or not there had been any voice improvements with the treatment (yes or no), which day the voice improved (1^st^ through the 7^th^) and report treatment-related side effects.

### Data analysis

The videolaryngoscopy recordings from each individual were shown to two otolaryngologists with experience in laryngology and in laryngeal exams (blinded as to which group the exam came from), the examiners were also blinded as to pre and post-treatment; and patient and exam dates were randomized.

The perceptive analysis of the vocal samples was carried out by three speech and hearing therapists - voice specialists, blinded as to and pre and post-treatment; and patient and exam date of the sustained [a] vowel were randomized. We utilized the GRBAS scale.

In order to establish the maximum phonation time, we utilized the mean value, in seconds, of the three samples of the vowel [a] as prolonged as possible (sustained in one single exhaling), in the patient's regular tone.

For acoustic analysis purposes, we utilized the mean values from the measures made by the DR Speech software, version 3.1, from the three samples of the mean utterance of the sustained vowel [e], before and after employing the treatments.

The results from the above analysis and the questionnaires were plotted on a table and submitted to statistical analysis.

## RESULTS

The statistical comparison of the variables: gender, age, profession, videolaryngostroboscopy data, and the auditory-perceptive assessments by the GRBAS scale and voice acoustics from the two groups before treatment, showed that the groups were similar in the beginning.

Videolaryngoscopy showed a significant reduction in hyperemia, edema and improvement in the muco-undulatory movement of the vocal folds after treatment. Edema reduction was statistically more significant in those patients treated with inhaled fluticasone (Table 1).

**Table 1:** Summary of the videolaryngostroboscopy and perceptive assessment (GRBAS) data before and after treatment with prednisolone and fluticasone and post-treatment comparison between the groups.

Variable	Ratio difference before × after		Comparison of the post-treatment assessment between the groups
	Prednisolone	Fluticasone	*p*-value
Hyperemia	-62.5	-62.5	1.000
Edema	-37.5	-81.3	0.012
Altered muco-undulatory movement	-50.0	-62.5	0.476
Full voice assessment	62.5	37.5	0.780
Roughness	37.5	31.3	1.000
Breathiness	56.3	43.8	O.780
Stress	18.8	12.5	1.000

The negative sign in the first three variables indicates a reduction in the trait.

In the perceptive assessment of the voice, both groups showed a significant improvement in voice quality, roughness and breathiness. Tension did not vary significantly between the two groups, and asthenia was not detected in any of the analyzed cases (Table 1).

The computerized acoustic analysis found a significant improvement in shimmer and NNE in those patients treated with oral prednisolone and in the jitter and NNE in those patients treated with inhaled fluticasone. The comparison of the post-treatment measures in both groups was not statistically significant (Table 2).

**Table 2:** Comparison of the acoustic assessment data before × after the *p*value in the treatments with prednisolone and fluticasone and between the groups after treatment.

Variable	Comparison between the values before × after (*p*-value)		Comparison between the groups after treatment (*p*-value)
	Prednisolone	Fluticasone	
Maximum phonation time (s)	1.000	0.164	0.876
Fundamental frequency (Hertz)	0.832	0.469	0.692
Jitter (%)	0.362	0.036	0.356
Shimmer (%)	0.044	0.205	0.083
Harmonic noise ratio: HNR (db)	0.438	0.121	0.089
Neutralized noise energy: NNE (db)	0.001	< 0.001	0.448

**Figure 1:**
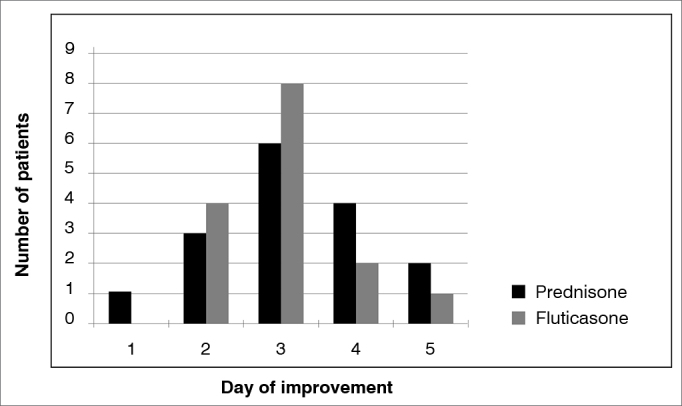
Day of improvement with the treatment reported by the patient.

In the questionnaires, all the patients reported improvements by the 5^th^ day of treatment. The logrank statistical analysis generated a *p* value = 0.627 indicating that there was no difference between the time to improve for patients using prednisolone and those who used fluticasone (Figure 1).

Of those patients who used prednisolone, four reported side effects (stomach pain and nausea) and one of the patients who used fluticasone reported an urge to cough upon drug administration.

## DISCUSSION

Acute dysphonia which limits verbal communication and prevents normal day-to-day activities is a common reason to seek medical care, especially in those patients who make a professional use of their voices. Before entering in the discussion part, we have to stress that in this paper we had two groups using the medication (inhalation and oral) and no control group without medication. This would enable us to answer how much of the development of these cases would be different in non-treated patients. Since this is a sample in which most individuals make professional use of their voices, the use of a placebo group brought about an important limitation in individual compliance towards participation. Patients in need of fast improvement did not feel comfortable with the possibility of being treated with a placebo. Having this difficulty, we decided to study these two treatment modalities, comparing the efficacy of the most-frequently mentioned treatment modalities in the literature.

Signs of acute inflammatory process (laryngeal hyperemia and edema, and alterations to the muco-undulatory movements of the vocal folds) were present in all dysphonic patients assessed by videolaryngostroboscopy in this study, before and after treatment. Seven days after the treatment with inhaled steroids (fluticasone) and oral steroids (prednisolone), there was an improvement in these signs in all the patients (100%), from both groups. However, edema reduction was greater in the group treated with the inhaled steroid.

The efficacy of oral steroids in the treatment of dysphonia caused by acute laryngitis is known in adults and has been described by some authors:

Mishra et al.^1^ mentioned oral and intramuscular steroids as the first-line treatment for singers with dysphonia caused by laryngitis, although they reported not finding any careful study on their efficacy in adults. These authors found only treatment studies based on subjective improvements and positive effects in pediatric patients, which we also found in our review of the literature.

Spiegel et al.^6^ suggested systemic steroids as powerful anti-inflammatory agents to treat dysphonia associated with acute laryngitis. Nonetheless, they reported that many otorhinolaryngologists use low doses (they mentioned 10mg of methylprednisolone), and that higher doses used for longer periods of time are more effective.

Sataloff et al.^7^ and Watts et al.^8^ reported cases of individuals making professional use of their voices, with acute laryngitis-related dysphonia, who needed to make an important speech, were successfully treated with oral steroids.

Klein et al.^2^ suggested the use of injectable or oral steroids in the treatment of laryngeal edema and acute vocal disorders.

Klassen^10^ and Pedersen et al.^11^ described the inhalation steroid as a better option in the anti-inflammatory drug-treatment for the airways, because of its high power as a topical agent and they stated that this is the ideal treatment for it produces a faster reduction in the inflammatory process, vascular permeability and mucosa edema. No study compared the two forms of treatment. In the literature, the studies are limited to case reports and citations, but without comparing the different forms.

The improvement in vocal quality seen in the global voice assessment parameters, roughness and breathiness, in both groups, after treatment was expected as a consequence to the improvement in the inflammatory process seen upon the videolaryngostroboscopy assessments. The stress parameter improved after treatment in both groups, but such improvement was not statistically significant in any of them. This result may be explained by the acute inflammatory process in the larynx. The stress is associated with the vocal effort caused by an increase in glottal adduction (adductor hyperfunction) which, in cases of acute laryngitis, may be limited because of discomfort and pain.

What was expected from the acoustic measures in the patients studied after a reduction in the inflammatory process was an increase in the mean values of the Fo, TMF and HNR, and a reduction in Jitter, Shimmer and NNE values. The expected happened in all the measures, in both treatments, when we analyzed the mean values. However, the changes were statistically significant only for the following variables: NNE in both groups, shimmer in the group with prednisolone and jitter in the fluticasone group.

The lack of total remission of the inflammation after treatment in some patients and a possible influence of a recent inflammatory process could justify the non-significant differences. Moreover, it is known that the acoustic assessment reliability depends on the quality of the recordings^24^. Patients with acute laryngitis have a major alteration in the quality of their voices associated with phonation irregularities, which makes it difficult to record their voices and causes these measures to be more prone to errors. In our study, the NNE detected a significant improvement after treatment in both groups assessed. NNE measures the sound wave noise, which is highly correlated with the auditory perception of dysphonia and roughness. Since it has the noise component as a basis, it is mentioned by Dejonckere^20^ and Pinho & Camargo^25^ as one of the most sensitive acoustic measures in the assessment of dysphonic voices.

Only a few authors have assessed acoustic measures in patients with laryngitis.

Plante et al.^26^ assessed the effectiveness of some objective acoustic analysis parameters during and after infectious laryngitis in four patients, and compared it with a control group having four healthy patients. The parameters investigated were: jitter, GNE (glottal to noise excitation) and the normalized error prediction (NEP). In this study, we used different programs and showed that it is possible to separate individuals with hoarseness from individuals with normal voice or after laryngitis recovery, using these three parameters. These authors discussed the variability of the parameters found among individuals of the same group and showed that this comparison is rather difficult. Each individual has his/her own phonation apparatus and physiology, and this introduces signal variations. Monitoring voice quality in the same individual is easier and more reliable, since it is the same phonation apparatus - and we also noticed this in our study. The individual comparative analyses showed significant statistical variations in our study before and after the two treatments (shimmer and NNE for prednisolone and Jitter and NNE for fluticasone), but these differences disappeared when we statistically analyzed the final measures in the two groups.

Ng et al.^27^ investigated the effect of acute laryngitis on the perceptive and acoustic aerodynamic measures in 11 patients with acute laryngitis before and after 7 to 10 days and compared with normal individuals. The fundamental frequency was reduced in the patients with laryngitis, suggesting an increase in the mass of the vocal folds during the course of laryngitis. The aerodynamic values were different in the cases of laryngitis, suggesting laryngeal hypofunction. The perceptive data showed the hoarseness of the patients with laryngitis - which was also observed in this study.

Watts et al.^13^ carried out a study with a patient with acute laryngitis, treated for six days with oral steroids, with the goal of assessing the effectiveness of treatment considering the acoustic measures on days 1, 3, 5 and 7. Their results showed a significant increase in the fundamental frequency, reduction in jitter, shimmer and in amplitude variability. These measures were not always linear, showing some oscillations which were contrary to what was expected in a few days - and we also found this in some of our patients. These oscillations happened, as per described above, because of difficulties to record the voice and the phonation instability inherent to patients with acute laryngitis.

Upon questionnaire assessment, all the patients studied in both groups reported improvements after treatment, correlated with what was observed in the assessments made by videolaryngostroboscopy and vocal assessment. They all reported improvements by the 5^th^ day of treatment, with a peak improvement on the 3^rd^ day, and there was no statistical difference in when the improvement happened between the patients who used the inhalation steroid (fluticasone) and oral (prednisolone). This data makes us rethink treatment duration. Would five days be enough?

Spiegel et al.^6^ reported that to treat acute laryngitis in adults, higher doses used for shorter periods of time are more effective, and they suggested 60 mg of prednisolone for 3 to 6 days. Watts et al.^8^ used 24 mg of methylprednisolone in a voice professional in the first day, and reduced it in 4 mg increments down to the 6^th^ day; and Franco & Andrus^3^ reported 16 mg/day of methylprednisolone, with a 4 mg reduction per day during 7 days. The ideal dose of the oral or inhaled medication is still uncertain, and we still need studies comparing the different therapeutic doses.

In our sample, four (25%) of the 16 patients who used the oral steroid (prednisolone) reported side effects (stomach pain and nausea) and one (6.3%) of the 16 patients who used inhaled steroids reported an urge to cough upon drug administration. These results are in agreement with the reports from Roland et al.^15^, who stated that the side effects of inhaled steroids, when compared to the systemic ones, are considered rare and milder. Spiegel et al.^6^ and Abaza et al.^17^ mentioned gastric irritation as one of most common adverse effects associated with oral steroids. The concomitant use of antacids, as per recommended by Abaza et al.^17^, during treatment with oral steroids is a suggestion to prevent gastric effects.

In none of the patients who used the inhaled steroid we noticed the laryngeal alterations described for asthmatic patients in chronic use of inhaled steroids^14^, ^15^, ^16^, ^17^, ^18^, ^19^. As per described by Watts^13^, we believe that these changes do not happen when low doses are used for just a few times a day and for a short period of time. The benefits surpass the potential risks, and inhaled steroids may be used with significant safety. The fear of adverse effects may stem from the lack of an effective and less risky treatment. We must stress that many patients, especially voice professionals, may have more frequent laryngitis episodes^3^ and a greater need to use steroids.

As to the inhaled steroid means of administration (inhaler type), we agree with Selroos et al.^23^ and Castro et al.^22^ who stated that the dry powder inhalers may be safer. They do not require propellants (which may cause irritation), since it is the patient's own breathing effort that causes drug dispersion in the airway. The patient must be instructed to wash the mouth after inhaling, in order to avoid side effects in this region, and he/she must also be instructed to make a medium intensity inhaling, because the goal is that the medication should reach the larynx with little impact.

We find it interesting to mention the review made by Mash et al.^28^ which, despite assessing studies from adult patients with asthma, had a similar goal to ours - to compare inhaled steroids (of different types) with oral prednisolone. This review assessed 1,285 studies and abstracts comparing inhaled steroids up to 2,000 mcg/day with oral steroids (specifically oral prednisone or prednisolone in up to 60 mg/day) in the treatment of adults with asthma. Only eight studies fulfilled the inclusion criteria. In six studies, prednisolone seemed to be as efficient as the inhaled steroid. In two trials, the inhaled steroid was more effective than prednisone. All inhaled steroid doses were more efficient than prednisolone in doses up to 60 mg in alternate days. The data reported on adverse effects were too variable to allow any comparison. A 30% incidence of patients receiving prednisolone in one study was reported, and there were no reports of adverse effects involving inhaled steroids.

And finally, we stress the recommendations from Spiegel et al.^6^ and Abaza et al.^17^ on the need for education, especially for voice professionals, in order to avoid abusing steroids. These drugs produce excellent clinical effect, with relatively few side effects when used for shorter periods of time and in low doses for the treatment of vocal disorders caused by inflammatory processes, but the risks of side effects may increase with frequent use.

## CONCLUSION

There was a significant improvement in the acute laryngitis vis-à -vis the assessments made, in all the patients studied, with both treatment modalities after 7 days. Treatment with the inhaled steroids (fluticasone) was significantly more effective in reducing the edema and produced fewer side effects than the treatment with oral steroids in this study.
